# How to Interpret a Positive *Campylobacter* PCR Result Using the BD MAX^TM^ System in the Absence of Positive Culture?

**DOI:** 10.3390/jcm8122138

**Published:** 2019-12-03

**Authors:** Thomas Gueudet, Marie Carole Paolini, Alice Buissonnière, Anne Trens, Jean Marc Rousée, Matthieu Lefranc, Lucie Bénéjat, Astrid Ducournau, Francis Mégraud, Emilie Bessède, Philippe Lehours

**Affiliations:** 1 Laboratoire SCHUH, BIO67-BIOSPHERE, 67000 Strasbourg, France; t.gueudet@bio67.fr (T.G.); a.trens@bio67.fr (A.T.); jm.rousee@bio67.fr (J.M.R.); 2 Laboratoire CBM25, 25000 Besançon, France; marie-carole.paolini@cbm25.fr (M.C.P.); matthieu.lefranc@cbm25.fr (M.L.); 3 CHU Pellegrin, Laboratoire de Bactériologie, CNR Campylobacters et Hélicobacters, 33076 Bordeaux, France; alice.buissonniere@chu-bordeaux.fr (A.B.); lucie.bruhl@chu-bordeaux.fr (L.B.); astrid.ducournau@chu-bordeaux.fr (A.D.); francis.megraud@chu-bordeaux.fr (F.M.); emilie.bessede@u-bordeaux.fr (E.B.); 4 INSERM UMR1053 BaRITOn, 33076 Bordeaux, France

**Keywords:** *Campylobacter*, culture, molecular diagnosis

## Abstract

The aim of this study was to evaluate, using two independent polymerase chain reaction (PCR) formats, the results of *Campylobacter* detection by the BD MAX^TM^ Enteric Bacterial Panel PCR (Becton Dickinson, Le Pont de Claix, France) in the absence of positive culture. A total of 77 samples found positive for *Campylobacter* on BD MAX^TM^ but negative by culture were studied. Upon reception, one in-house real-time-PCR for *Campylobacter sp.* and a PCR with the RIDAGENE Bacterial Stool Panel (r-biopharm, Darmstadt, Germany) were performed. The data obtained using these two PCR formats were evaluated with respect to the cycle threshold (Ct) and fluorescence intensity values (FI) obtained on BD MAX^TM^. Ct and FI values were also obtained for 80 positive *Campylobacter* cases by culture. Among the 77 samples, 33 were positive with the two PCRs, and 37 remained negative. For the 33 double-positive PCRs samples, the Ct values obtained on BD MAX^TM^ were lower than 30 in 93.9%, and FI > 2000 for 97% of cases. For the 37 double-negative PCRs samples, the Ct values obtained on BD MAX^TM^ were <30 in only 18.9%, however FI were >2000 for 40.5% of cases. Positive culture cases had Ct values < 30 in 96.2% and FI > 2000 in 98.8%. We showed that the Ct values obtained on BD MAX^TM^ can help to interpret the results. Almost 96% of the *Campylobacter* *sp.* cases detected by culture or with the two reference PCRs positive showed a Ct value on BD MAX^TM^, meaning that stools detected as positive with BD MAX^TM^ and having a Ct > 30 may be false positives.

## 1. Introduction

Campylobacters are among the most prevalent bacteria involved in acute diarrhea [1]. The culture of *Campylobacter sp.* can unfortunately be falsely negative in 10% to 30% of cases [2] due to preanalytical and analytical factors. Clinical laboratories equipped with syndromic polymerase chain reaction (PCR) systems in the context of a bacterial infectious diarrhea diagnosis are tempted to use only selective agar for the pathogen(s) detected by PCR. The French National Reference Center for Campylobacters and Helicobacters (NRCCH) set up a collaboration with two diagnostic laboratories participating in its surveillance network, located in the French departments 25 (Doubs) (Laboratory CBM25) and 67 (Bas-Rhin) (Laboratory Bio67), using the BD MAX^TM^ Enteric Bacterial Panel PCR (Becton Dickinson, Le Pont de Claix, France) as a screening method before culture. These two laboratories perform *Campylobacter* culture only on stools that are detected positive on the BD MAX^TM^, leading to a negative culture in a few cases. Thus, the objective of the study was to verify the positive *Campylobacter sp.* status of the stools negative by culture for the period from 4 January to 23 August 2018 and from 18 December 2017 to 30 June 2018 for Laboratories CBM25 and Bio67, respectively.

## 2. Materials and Methods

The two laboratories sent 77 samples to the NRCCH (53 Cary Blair transport medium tubes previously inoculated with rectal swabs received from Bio67 and 24 stools from CBM25) (patients with a sex ratio 0.52 and a mean age 46.9 year ± 24.9) that were found positive for *Campylobacter sp*. on BD MAX^TM^ but negative by culture (see Table 1 for culture conditions in each laboratory).

The presence of diarrhea and/or abdominal pain was reported for only 25 cases (32.5%). For the 52 remaining cases, no clinical symptoms were reported by the two participating laboratories. The aspect of the stools was reported for 48 samples (62.3%), among them 28 were loose stools, 15 liquid, 3 glairy and 3 bloody. Only 4 of the 77 patients (5.2%) were already on antibiotic treatment at the time of stool collection. Simultaneous detection of other enteropathogens was found for 5 patients only (6.5%): 1 *Yersinia sp.,* 3 pathogenic *Escherichia coli* (1 enterotoxinogen, 2 enterohemorragic) and 1 threadworm.

All of the samples were stored at −80 °C and transported to the NRCCH on dry ice. Upon reception, samples were cultured directly on Campylosel agar (bioMérieux, Marcy l’Etoile, France) (50 µL of Cary Blair transport medium or 50 µL from stools diluted in 9 mL of saline water) and incubated for 72 h at 35 °C in a microaerobic atmosphere, as previously described [3,4], and after enrichment in Preston broth inoculated with either a nut of stools or 100 µL of liquid stool or Cary Blair transport medium (24 h at 35 °C before subculturing on trypticase soy agar enriched with horse blood (bioMérieux)). They did not undergo other freeze–thaw cycles.

DNA extraction was performed on the stored samples (500 µL of Cary Blair transport medium or 1 mL of a stool suspension in GXT Stool Stabilizer provided with the GXT Stool Extraction kit) (Hain Lifesciences, Nehren, Germany) at the NRCCH using an Arrow automation (NorDiag, Diasorin, Saluggia, Italy). Real-time PCR was then carried out to identify *Campylobacter jejuni, Campylobacter coli* and *Campylobacter fetus* using a LightCycler 2.0 (Roche Life Science, Indianapolis, IN, USA), as previously described [5]. Further testing was performed with the commercially available RIDAGENE Bacterial Stool Panel (r-Biopharm, Darmstadt, Germany) on CFX96 (Biorad, Marnes-la-Coquette, France) according to the manufacturer’s protocol.

The data obtained using these two PCR formats were evaluated with respect to the cycle threshold (Ct) and fluorescence intensity values (FI) obtained on BD MAX^TM^. The total number of BD MAX^TM^ *Campylobacter* sp. positives for each laboratory is indicated in Table 1. The Ct and FI values obtained for 80 positive *Campylobacter sp.* cases by culture were also provided by the two laboratories.

Statistical analyses were performed with GraphPad Prism 5.01 (GraphPad Software, Inc., La Jolla, CA, USA). The Mann–Whitney test was used as a nonparametric test to compare the distributions of two unmatched groups. Differences were considered significant when *p* was inferior to 0.05.

## 3. Results

The activity report and the analytical conditions used by the two laboratories for culturing *Campylobacters* are summarized in Table 1. In these laboratories the level of negative culture for a positive *Campylobacter* PCR detection was 10.6% and 12.5% for CBM25 and Bio67 laboratories, respectively (Table 1). During the time periods considered, the ratio of positive *Campylobacter sp.* to positive PCR versus *Salmonella sp.* was 423/7 and 227/13 for Bio67 and CMB25, respectively. These results confirm the wide prevalence of *Campylobacter sp*. in acute community infectious diarrhea.

Culture of the 77 samples was also negative at the NRCCH. Thirty-three of the 77 samples (7 from stools and 26 from Cary Blair transport media) (42.9%) were positive with the two PCR formats tested (double-positive PCR cases) (30 *C. jejuni* and 3 *C. coli*), and 37 samples (15 from stools and 22 from Cary Blair transport media) (48%) remained negative with the two PCRs (double-negative PCR cases). Six samples were PCR positive with the RIDAGENE Bacterial Stool Panel only (7.8%) and 1 sample (1.3%) was positive with the in-house PCR only. No significant difference of Ct values was found between the stools and Cary Blair transport media (Figure S1).

For the 33 double-positive samples, diarrhea was noticed in only 3 cases whereas no symptom was reported in the 30 remaining cases. Eight of these samples corresponded to loose stools, 1 glairy, 2 bloody, 8 liquid, and for the 14 remaining samples, the stool aspect was not reported.

For the 37 double-negative samples, diarrhea and/or abdominal pain were present in 17 patients whereas no symptom was reported in the 20 remaining cases. The majority of these samples corresponded to loose stools (*n* = 17), 1 glairy, 5 liquid, and for the 14 remaining samples, the stool aspect was not reported. None of the 37 double-negative samples corresponded to patients that were under antibiotic treatment.

**Figure 1:**
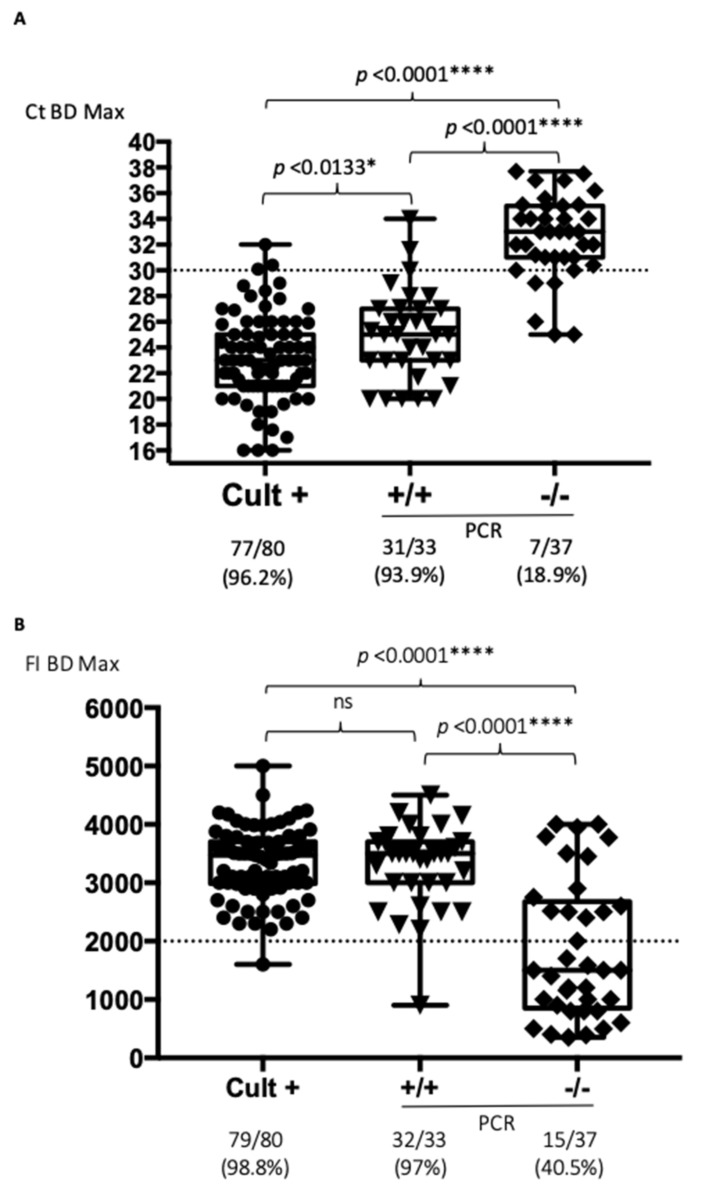
Stratification of the BD MAX^TM^ results according to culture results and *Campylobacter sp.* detection by independent PCR formats (* *p* < 0.05, and **** *p* < 0.0001, ns: non-significant). (**A**) Data analyzed according to the Ct values obtained on BD MAX^TM^; (**B**) Data analyzed according to the fluorescence intensity (FI) obtained on BD MAX^TM^. Cult+: positive *Campylobacter* cases; +/+: culture negative cases positive by two independent PCRs; -/-: double-negative cases. The doted lines correspond to the proposed cut-off for Ct or FI values.

The Ct values initially obtained for *Campylobacter* detection using the BD MAX^TM^ system for 31 of the 33 double-positive PCR samples were lower or equal to 30 cycles (93.9%), versus only 7 of the 37 double-negative PCR cases (18.9%) (*p* < 0.0001) (Figure 1A).

As a comparator, Ct values were also ≤30 in 77 out of 80 positive culture cases (96.2%) (Figure 1A). It therefore seems that the Ct values may be a good indicator of the presence of *Campylobacter sp.* detectable by culture or at least confirmed by two independent PCR formats. For the 80 positive culture cases, diarrhea was noticed in 58 cases; 1 patient had abdominal pain, whereas unfortunately no symptom was reported in the 21 remaining cases. Most of them (*n* = 34) corresponded to liquid stools, 19 loose stools, 8 glairy samples; 2 bloody stools and for the 17 remaining samples, the stool aspect was not reported.

The same analyses were performed based on the FI values obtained on the BD MAX^TM^ system. The FI values were higher than 2000 units in 32 of the 33 double-positive cases (97%), but also in 15 of the 37 double-negative cases (40.5%) (*p* < 0.0001) (Figure 1B). Positive culture cases had an FI > 2000 in 79 out of 80 samples (98.8%). It therefore seems that FI values are less discriminatory than Ct values (Figure 1B). No significant difference of FI values was found for the double-negative cases between the stools and Cary Blair transport media, whereas FI values were significantly higher in the 7 double-positive stools compared to the 22 Cary Blair transport media (*p* = 0.0484) (Figure S1).

## 4. Discussion

The BD MAX^TM^ Enteric Bacterial Panel PCR clearly improves the diagnosis of *Campylobacter sp.* infections by enabling the laboratories to deliver fast and targeted results to their clinicians [6,7]. Syndromic approaches now replace the traditional research by culture. Culture is performed only in the case of a positive BD MAX^TM^ result. Thus, technicians examine only agar plates in which they are supposed to find the pathogen detected by PCR, which probably reinforces their vigilance in identifying suspicious colonies. This selective culture is, however, at the expense of pathogens not targeted by the chosen syndromic PCR kit.

The BD MAX^TM^ PCR gives relevant results concerning the detection of Campylobacters. Without a perfect gold standard such as a positive *Campylobacter sp.* culture, only double-positive cases by two independent PCRs could be considered as true *Campylobacter sp.* positives in case of negative culture [2,8,9]. We did not find a perfect correlation between the *Campylobacter* PCR results obtained on BD MAX^TM^ and the two independent PCR formats. Several factors could explain this: the extraction of DNA is not identical, the targets used are different, the detection limits can vary between the methods (see below), and the DNA may have been degraded by the presence of DNAse in the stool sample. All of these factors should be evaluated in a future study. According to the manufacturer, the mean detection limit of *Campylobacter* detection by the RIDAGENE PCR is 2.1 × 10^4^ CFU/mL and 8.5 × 10^5^ CFU/mL for *C. jejuni* and *C. coli*, respectively (manufacturer data). For the BD MAX^TM^ system the mean detection limit for *C. jejuni* is around 6.3 × 10^3^ CFU/mL, and 1.5 × 10^3^ CFU/mL for stools and Cary Blair, respectively. For *C. coli*, it is around 1.4 × 10^4^ CFU/mL, and 8.2 × 10^3^ CFU/mL for stools and Cary Blair, respectively (manufacturer’s data). Our in-house *gyrA* PCR was originally designed to identify *Campylobacter* [5]. Its ability to detect *Campylobacter* in stools has never been evaluated.

The nature of the samples tested in the present study (either stools or swabs in Cary Blair transport media) has no influence on the *Campylobacter sp.* detection, confirming that there is no influence of inoculum or inhibitory issues as suggested by others [6]. Even if the preanalytical conditions were different between the two laboratories, the Ct values were significantly lower in the positive stools in culture. The most likely hypothesis is that these patients had a more abundant bacterial load in their digestive tract. This has already been suggested by the data published by Wholwend et al., where the authors showed that the median Ct values of culture-positive specimens were significantly lower than those of culture-negative specimens. [10].

Nonetheless, PCRs only obtained using the BD MAX™ remained low, with 5.7% (37 samples) of all 650 samples tested in the two laboratories during the time period of the present study.

A limit of this study is its retrospective design, which did not allow to obtain clinical data for 52 of the 77 samples included in the analyses. Even if there were more loose stools in the double-negative cases compared to the double-positive cases, it could not be a good indicator of the presence of *Campylobacter sp*.

However, this study shows that 95.6% (108/113) of the *Campylobacter sp.* cases detected by culture or with the two reference PCRs were also positive on BD MAX^TM^ with a Ct value ≤ 30; hence, stools that were positive on BD MAX^TM^ with a Ct > 30 may possibly be false positives. In our study, this cut-off had a sensitivity of 93.9% and a specificity of 81%. The fact that the Ct threshold proposed in our study was valid for two independent laboratories is representative of its robustness. The integration of a quantitative control for adjusting Ct values to chromosomal equivalents could be of major interest.

## 5. Conclusions

In conclusion, it could be interesting to propose that laboratories using the BD MAX^TM^ strategy for *Campylobacter sp.* diagnosis before culture, include a comment concerning the clinical interpretation and the therapeutic management of positive cases with Ct values > 30. This comment should alert the clinician of the risk of having no positive *Campylobacter sp.* culture, in which case the clinician should reconsider the need of giving empiric antimicrobial therapy. However, these results could be confirmed by a prospective study.

We recommend that this type of analysis should be carried out for other enteric pathogens and for other PCR formats.

## Figures and Tables

**Table 1:** Activity report of the two laboratories participating in the study.

	Lab. CBM25	Lab. Bio67
Time period	01/04 to 08/23/2018	12/18/2017 to 06/30/2018
No. of samples	2076	4866
No. of *Campylobacter* sp. + BD MAX^TM^ PCR	227	423
No. of *Campylobacter* sp. isolated by culture	203	369
% of culture-/*Campylobacter* sp.+ BD MAX^TM^ PCR	(24/227) 10.6%	(53/423) 12.5%
Selective agar plates and culture conditions	Butzler (10 µL) 5 days in a jar	CASA (20 µL) 3 days in a sachet

No.: number; Lab: laboratory. Butzler agar plates are commercialized in France by Biorad (Marnes La coquette, France). CASA is a chromogenic medium commercialized by bioMérieux (Marcy L’Etoile, France).

## Supplementary Materials

The following are available online at https://www.mdpi.com/2077-0383/8/12/2138/s1, Figure S1: Stratification of Ct intensity and fluorescence values according to the nature of the samples (* *p* < 0.05, ns: non-significant).
