# Supplementary material for: How to Interpret a Positive Campylobacter PCR Result Using the BD MAXTM System in the Absence of Positive Culture?
Source: J Clin Med. 2019 Dec 3;8(12):2138. doi: 10.3390/jcm8122138 (PMC6947629; doi:10.3390/jcm8122138)
Supplement: Supplementary file 1 [file jcm-08-02138-s001.pdf]

A

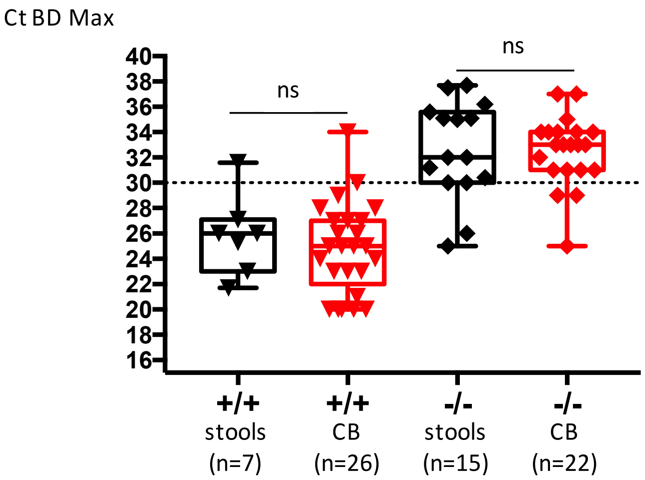

B

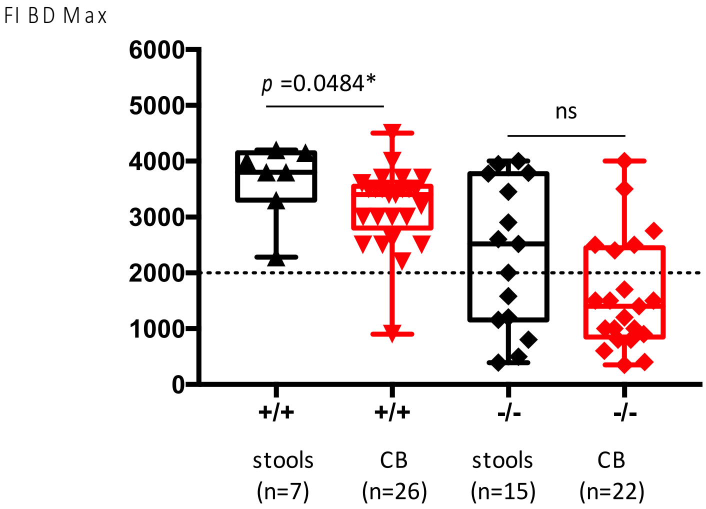

Figure S1. Stratification of Ct intensity and fluorescence values according to the nature of the samples (* $p < 0.05$, ns: non-significant). **(A)** Data analyzed according to the Ct values obtained on BD MAX™; (**B**) Data analyzed according to the fluorescence intensity (FI) obtained on BD MAX™. +/+: culture negative cases positive by two independent PCRs; -/-: double negative cases. The doted lines correspond to the proposed cut-off for Ct or FI values. CB: Cary Blair (in red). Stools in dark.
